# ChatGPT opens a new door for bioinformatics

**DOI:** 10.15302/J-QB-023-0328

**Published:** 2023-06

**Authors:** Dong Xu

**Affiliations:** Department of Electrical Engineering and Computer Science, Christopher S. Bond Life Sciences Center, University of Missouri, Columbia, MO 65211, USA

ChatGPT is an artificial intelligence (AI) system that can perform sophisticated writing and dialogs after learning from vast amounts of linguistic data. The success of ChatGPT is phenomenal. AI-based human-machine language interaction has been at the center of AI competition in recent years. The major players in this game have been Google, Meta, and OpenAI. Google was in the best position from the outset, given its invention of Transformer (the cornerstone of all cutting-edge language models) and its significant edge in reinforcement learning. Yet, Google’s efforts in this area were rather diffusing. It kept generating language model variants with incremental innovations but failed to reach the next level. Meta has a strong AI team, including many top AI researchers in the world. Nevertheless, their faith in self-supervised learning to solve human-machine interaction did not deliver high-impact success. Conversely, OpenAI, with a small team, stayed focused on a single product line (GPT, including its latest release of GPT-4). It moved in the right direction of using human input to “*align*” the language model based on the Reinforcement Learning from Human Feedback (RLHF) approach. The fact that OpenAI ultimately prevailed in this game shows that the model *alignment* to human labeling through supervised and reinforcement learning is critical for human-machine interaction. However, a chatbot’s actions rely heavily on cues (*prompts*) provided by human operators. To properly utilize ChatGPT’s capabilities, *prompts* to instruct or mentor the chatbot must be carefully designed to get valuable, valid, and robust responses. This process becomes another “*alignment*” problem of using *prompt* engineering to best probe ChatGPT’s knowledge graph for best serving users’ needs.

ChatGPT has sparked tremendous interests in many fields. It has potential uses in facilitating and tutoring programming-heavy data analysis, like bioinformatics. However, the investigation of ChatGPT in biology and medicine is less extensive than in other domains. As of March 4, 2023, ChatGPT had 5380 appearances in Google Scholar’s general publication database, but just 21 and 8 in the bio-specific preprint repositories medRxiv and bioRxiv, respectively. Within the 5380 articles in Google Scholar, 75 articles mentioned ChatGPT and bioinformatics simultaneously. In contrast, 3540, 2560, and 2550 concurrently incorporated ChatGPT, and education, economics, or law, respectively. Such statistics reflect the general trends in different fields consistently, but among the 75 articles in bioinformatics, only one editorial focused on ChatGPT’s usage in bioinformatics [1], while others just passingly mentioned it.

Meanwhile, ChatGPT has demonstrated excellent promise in performing intricate biomedical tasks. For instance, ChatGPT passed the United States Medical Licensure Test with a 60% accuracy score without the assistance of human trainers [2]. ChatGPT has several benefits for bioinformatics: (i) Given the multidisciplinary nature of bioinformatics, ChatGPT can assist bioinformatics researchers in staying current on a variety of relevant research subjects. (ii) It can facilitate complex tasks involving substantial biodata, particularly for time-sensitive biomedical applications. (iii) ChatGPT may be tailored to suit diverse bioinformatics jobs with its strong domain-adaptation capabilities. (iv) ChatGPT variants can generate effective language descriptions for nucleotides, proteins, and chemical compounds to perform downstream bioinformatics tasks. (v) ChatGPT can be used to mine the biomedical knowledge graph [3] (our research team has also demonstrated that ChatGPT can effectively predict gene relationships).

Thanks to its remarkable conversational and programming abilities, ChatGPT holds great promise for helping students overcome programming hurdles. Recently, Dr. Gangqing Hu and his collaborators introduced the OPTIMAL (Optimization of Prompts Through Iterative Mentoring and Assessment) model for leveraging ChatGPT to assist in programming-heavy data analysis in bioinformatics [4]. Inspired by adaptive learning in education, OPTIMAL facilitates chatbot-aided data analysis through iterative steps to improve communication with a chatbot, better *align* data analysis expectations, and enhance students’ learning outcomes. In this paradigm, students evaluate the research question, computational task, analysis methods, and anticipated outputs. They are given instructions on how to design a *prompt* for the data analysis assignment at varying levels of detail. Students then input the *prompt* to ChatGPT to generate and execute the code. If error messages appear after running the code, students review them and determine how to proceed, such as instructing ChatGPT to revise the code or manually debugging it. Iterations of this procedure continue until the code is free of errors. After that, students review the above communication process and the code to update the initial *prompts* for another round of analysis. As a proof of concept, the study has demonstrated the model’s effectiveness in three bioinformatics case studies, *i*.*e*., next-generation sequencing analysis, molecular evolution, and computer vision. It shows the potential to streamline the iteration process and enhance students’ critical thinking and evaluation skills.

The OPTIMAL model provides an excellent example for many possible ChatGPT applications in bioinformatics education. In addition to interactive learning, tutoring, and data analysis support as demonstrated in this model, ChatGPT can also be used to (i) survey state-of-art development of a bioinformatics topic; (ii) produce step-by-step tutorials and suggest appropriate learning resources and activities; (iii) map bioinformatics concepts and their interrelationships; and (iv) plan a bioinformatics curriculum. Nonetheless, the use of ChatGPT in biomedical research is still new. As pointed out by the authors [4], the effectiveness of the data analysis heavily relies on *prompts*. Mentoring or instruction techniques for ChatGPT are still *ad hoc*, and additional research is required to build design principles for better *aligning* the expected outcomes (again, the key is *alignment*)! In addition, the chatting process requires explicit and implicit prerequisite knowledge to have a reasonable probability of converging to a satisfying end point; otherwise, it may be trapped in an unending cycle or arrive at incorrect conclusions. It would be beneficial for the chatbot to identify students’ knowledge gaps from their *prompts* and guide them to learn related concepts. Furthermore, extensive usability tests in a fashion like clinical trials are required to fully characterize the benefits, drawbacks, and areas of improvement for systematically incorporating ChatGPT into the pedagogy of bioinformatics education.

In summary, the OPTIMAL model pioneered chatbot-aided bioinformatics data analysis and tutoring by employing a series of iterative steps to improve student learning outcomes. Such a strategy may help students develop their coding and analysis abilities, as well as their critical and creative thinking. The strategy can probably go beyond the classroom and into a lifelong learning experience. Like many other fields, ChatGPT will also gain ground in bioinformatics, from education and literature mining to data analysis and method development.
